# Prospects, challenges and perspectives in harnessing natural selection to solve the ‘varroa problem’ of honey bees

**DOI:** 10.1111/eva.13533

**Published:** 2023-02-21

**Authors:** Matthieu Guichard, Benjamin Dainat, Vincent Dietemann

**Affiliations:** ^1^ Swiss Bee Research Centre Agroscope Bern Switzerland; ^2^ Department of Ecology and Evolution, Biophore, UNIL‐Sorge University of Lausanne Lausanne Switzerland

**Keywords:** colony losses, Darwinian beekeeping, genotype–environment interaction, honey bee, natural selection, *Varroa destructor*

## Abstract

Honey bees, *Apis mellifera*, of European origin are major pollinators of crops and wild flora. Their endemic and exported populations are threatened by a variety of abiotic and biotic factors. Among the latter, the ectoparasitic mite *Varroa destructor* is the most important single cause behind colony mortality. The selection of mite resistance in honey bee populations has been deemed a more sustainable solution to its control than varroacidal treatments. Because natural selection has led to the survival of some European and African honey bee populations to *V. destructor* infestations, harnessing its principles has recently been highlighted as a more efficient way to provide honey bee lineages that survive infestations when compared with conventional selection on resistance traits against the parasite. However, the challenges and drawbacks of harnessing natural selection to solve the varroa problem have only been minimally addressed. We argue that failing to consider these issues could lead to counterproductive results, such as increased mite virulence, loss of genetic diversity reducing host resilience, population collapses or poor acceptance by beekeepers. Therefore, it appears timely to evaluate the prospects for the success of such programmes and the qualities of the populations obtained. After reviewing the approaches proposed in the literature and their outcomes, we consider their advantages and drawbacks and propose perspectives to overcome their limitations. In these considerations, we not only reflect on the theoretical aspects of host–parasite relationships but also on the currently largely neglected practical constraints, that is, the requirements for productive beekeeping, conservation or rewilding objectives. To optimize natural selection‐based programmes towards these objectives, we suggest designs based on a combination of nature‐driven phenotypic differentiation and human‐directed selection of traits. Such a dual strategy aims at allowing field‐realistic evolutionary approaches towards the survival of *V. destructor* infestations and the improvement of honey bee health.

## 1 INTRODUCTION

Several decades after the start of its invasive spread, the ectoparasitic mite *Varroa destructor* (Anderson & Trueman) remains the most detrimental biotic agent affecting the health of honey bees (*Apis mellifera* L.) of European origin (Rosenkranz et al., 2010; Traynor et al., 2020). This mite has eradicated most wild and feral *A. mellifera* populations in Europe and the regions where this species was introduced (Jaffé et al., 2009). Given the large economic value of this main managed pollinator and hive product provider (Brosi et al., 2017), *V. destructor* is ranked fifth in the list of the most socio‐economic and environmentally costly animals or plants invaders in Europe (Nentwig et al., 2018). This mite feeds on adult and immature honey bees and vectors deadly viruses (Bowen‐Walker et al., 1999; Dainat et al., 2012), often leading to colony death within a few years (Büchler, 1994; Korpela et al., 1992). The respective roles of the mite and of its accompanying viruses in colony mortality are not yet clearly understood, but their interplay is often referred to by beekeepers and scientists as the ‘varroa problem’ (Dietemann et al., 2012). Controlling the mite population in the colonies is recommended to prevent mites and their associated viruses from spreading (Jack & Ellis, 2021; Rosenkranz et al., 2010). To ensure the survival of their stock, beekeepers need to apply varroacidal treatments, often repeatedly throughout the year (Hernandez et al., 2022; Oberreiter & Brodschneider, 2020; Rosenkranz et al., 2010). There is a consensus that a more sustainable solution could be achieved by breeding honey bee lineages resistant to the parasite (Dietemann et al., 2012; Mondet, Parejo, et al., 2020). The occurrence of large populations of African honey bee subspecies and of some small European populations surviving infestations by *V. destructor* without the need for treatment (Guichard et al., 2020; Le Conte et al., 2020; Martin & Medina, 2004; Moritz & Hänel, 1984; Nganso et al., 2018; Strauss et al., 2016) have fuelled the hope that natural selection can solve the varroa problem. Accordingly, in recent years, several articles have argued that harnessing ecological and evolutionary principles by letting natural selection operate could promote honey bee health, especially for susceptible populations of European honey bees (Brosi et al., 2017; Neumann & Blacquière, 2017; Seeley, 2017). It has also been argued that this approach could be more relevant and effective than human‐directed selection for resistance traits to provide stock of populations surviving *V. destructor* infestations (hereafter simply designated as ‘surviving’) (Brosi et al., 2017; Neumann & Blacquière, 2017; Seeley, 2017), to the point that these approaches have been considered as mutually exclusive (Neumann & Blacquière, 2017).

**FIGURE 1:**
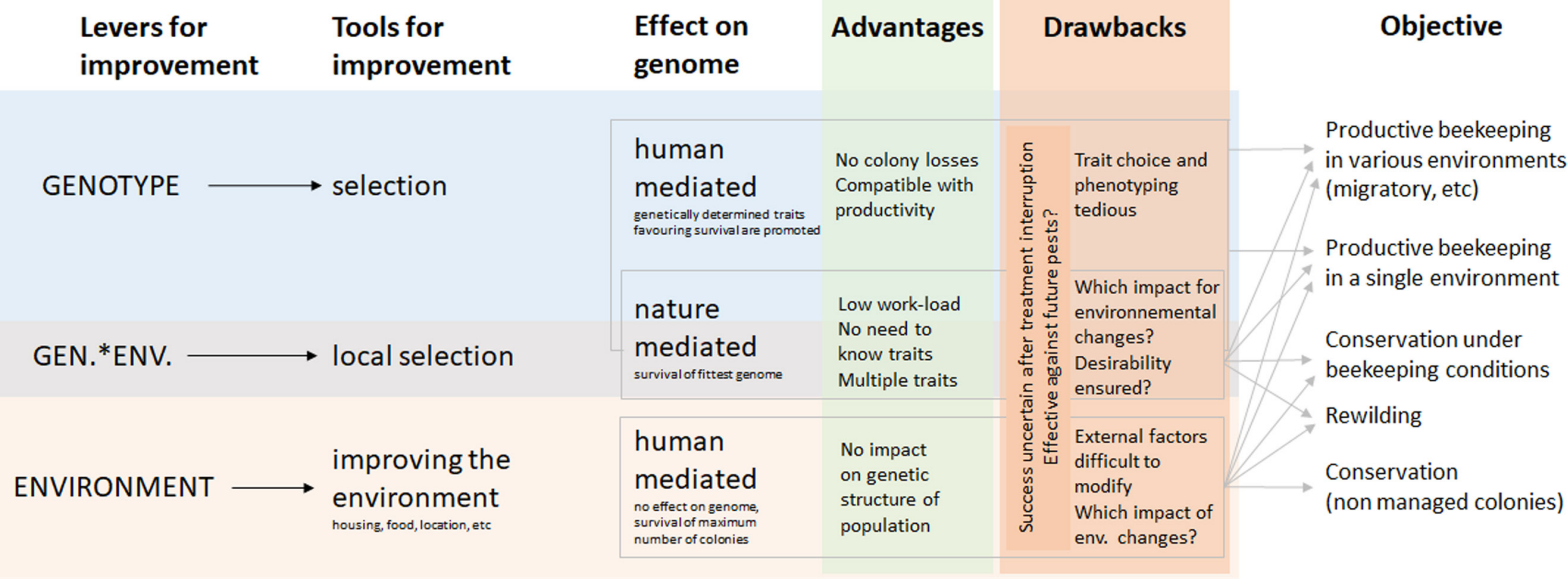
Principles of natural and human‐directed selection with levers and tools for improvement, origin of the effects on genome, if any, and advantages and drawbacks. Several objectives were considered. GEN.*ENV., Genotype–Environment interaction.

Harnessing natural selection to solve the ‘varroa problem’ is advantageous for several reasons (Figure 1). The pressure exerted by the parasite on honey bee colonies in the absence of treatments roots out the susceptible genotypes without the need to know the complex underlying mechanisms, which is required to improve the success of human‐directed selection of survival traits (Guichard et al., 2020). Thus, natural selection‐based programmes (see the Glossary) appear to be less laborious for beekeepers and breeders, are deemed to have higher chances of success (Neumann & Blacquière, 2017; Seeley, 2017) and are gaining traction among a growing number of stakeholders eager for nature‐based approaches against this parasite. The theoretical and practical drawbacks and challenges of natural selection‐based approaches (Figure 1) have only been briefly mentioned in the literature (Brosi et al., 2017; Seeley, 2017); however, there is a need to consider them in depth to assess the perspectives offered and challenges posed by natural selection‐based approaches. Failing to do so could lead, after several years of selection, to counterproductive outcomes, such as increased mite virulence, loss of genetic diversity reducing host resilience, sudden population collapses and wasted resources.

Harnessing natural selection to increase the survivability of honey bee colonies infested by *V. destructor* can contribute to three main objectives: productive beekeeping, local honey bee conservation or rewilding (see the Glossary), which may vary according to the beekeepers' profiles (Blacquière, Boot, & Calis, 2019; Kahane et al., 2022; Underwood et al., 2019). Each objective requires colonies that show different properties. Commercial beekeeping benefits from gentle, highly productive stock with low swarming behaviour, for example. These traits are not required for the conservation of local subspecies and ecotypes or for rewilding towards the reestablishment of free‐living honey bee populations in their natural environment. For these two objectives, only the long‐term ability of the population to survive in the local environment is required; for the latter, the ability to survive without supplementary feeding by beekeepers is crucial. To date, these distinct properties have not been considered when discussing programmes to harness natural selection to generate honey bee stocks surviving *V. destructor* infestations.

Our main aim was to assess the perspectives offered by natural selection approaches. These perspectives can be derived from past achievements and from theoretical and practical challenges that still need to be overcome. To determine whether and to what extent natural selection has already been successfully harnessed to solve the ‘varroa problem’, we first reviewed the available literature for the reported outcomes, context and strategies of such programmes. Second, we compiled both the advantages and disadvantages of natural selection approaches to better balance the strengths of this approach with the challenges it can bring. Finally, to minimize unwanted outcomes and optimize the probability of success of natural selection‐based selection programmes, we propose strategies adapted for each of the three possible objectives: productive beekeeping, conservation and rewilding. These strategies associate the benefits of both a nature‐driven phenotypic differentiation of colonies (the fittest develop well, while the weakest stagnate and are removed or treated before collapse) and human‐directed selection of survivors with desirable traits, both using conventional breeding tools. The validity and success of programmes following such strategies should be verified by scientific monitoring of progress in the field to allow for optimal progress and solutions.

## 2 PROSPECTS FOR HARNESSING NATURAL PROCESSES TO SOLVE THE ‘VARROA PROBLEM’

The prospects for harnessing natural processes to select honey bee stock surviving despite infestation with *V. destructor*, as well as the best manner to reach this goal, could be derived from existing or past programmes in which beekeepers and breeders followed this strategy. Two approaches have been used to generate surviving lineages. The fastest manner to obtain stock that could survive *V. destructor* infestation is to import colonies from regions where the host and parasites have already reached a coevolutionary equilibrium, such as those of African subspecies of *A. mellifera* (Boecking & Ritter, 1993; Osterlund, 1991, 2001; Ritter et al., 1990). Colonies were also imported from the far‐eastern region of Russia (Primorsky), where the *A. mellifera* populations had been exposed to *V. destructor* soon after its host shift (Crane, 1978; Traynor et al., 2020), which could have provided them with more time to adapt to the parasite than populations infested later (Danka et al., 1995). The second approach consisted of using a local population exposed to the selection pressure of *V. destructor* by interrupting varroacidal treatments. This approach has the advantage of allowing the retention of locally adapted traits, which is suggested by the higher survival of local rather than translocated stock (Büchler et al., 2014; Kovačić et al., 2020). Thus, genotype–environment interaction mechanisms can contribute to colony survival, in addition to the additive genetic effects of the mechanisms favouring survival to *V. destructor* infestations (Figure 1). To determine how successful these approaches were, we reviewed the literature for their genesis, for evidence that the strategies followed led to surviving populations and to determine how widely they are available to beekeepers.

### 2.1 Were imports of *V. destructor*‐resistant honey bee stock successful?

Honey bee lineages derived from imported stock (Primorsky and *A. m. intermissa* from Tunisia) have shown lower mortality and mite infestation rates compared with local controls in their new environment (de Guzman et al., 2007, 2019; Kefuss et al., 2004; Rinderer, de Guzman, Delatte, Stelzer, Lancaster, et al., 2001a; Tarpy et al., 2007; Thrybom & Fries, 1991) and need fewer or no varroacidal treatments to survive (Ward et al., 2008; Webster, 2019). However, importing resistance can only be deemed successful if the original population clearly survives infestations. Despite low infestation rates in their region of origin (Danka et al., 1995), the resistance level of the Primorsky *A. mellifera* population remains unclear. The colonies were treated against the parasite by local beekeepers, and few wild or feral colonies were observed in the region (Danka et al., 1995), which did not unambiguously support this population's survivability. Similarly, the resistance status of the *A. m. intermissa* population (Boecking & Ritter, 1993; Ritter et al., 1990) that has been used to constitute a surviving commercial stock in Southern France (Kefuss et al., 2004) is unclear given reports of the need for varroacidal treatments in the original distribution range of this subspecies (Adjlane et al., 2015).

If not because of the resistance traits of their original population, how can the good performance of these lineages be explained? The properties of the Primorsky stock could be the result of selecting low *V. destructor* infestation rates that the stock was subjected to after its import (Rinderer et al., 1999, 2000; Rinderer, de Guzman, Delatte, Stelzer, Lancaster, et al., 2001a; Rinderer, de Guzman, Delatte, Stelzer, Williams, et al., 2001b). Unless the survival traits were maintained via the female lineage in the descendants of the *A. m. intermissa* stock, survival would unlikely be of genetic origin. Indeed, survivability is maintained even under open mating with the sexuals of the susceptible‐treated neighbouring populations in the new environment (Bandi, 2019; Kefuss et al., 2004), which is expected to lead to frequent losses in the surviving population because of the introgression of susceptible genes.

Several studies have suggested that the disintegration of local adaptation when naturally surviving genotypes are translocated leads to a loss of or to a lower survivability (Berg et al., 2002; Büchler et al., 2014; Correa Marques et al., 2002; Koeniger et al., 1995; Kovačić et al., 2020; Meixner et al., 2015; Rosenkranz, 1999), adding to the unpredictability of the success of imports.

Because of the current uncertainty in the ability of original populations to survive *V. destructor* infestations without treatments and in the genetic basis of the underlying traits, there is no clear evidence that queen imports are an effective way to obtain resistant stock. This calls into question whether this approach can lead to a high probability of success under most circumstances. Importing foreign honey bees has also been tied to many drawbacks, such as introgression of foreign genes in local populations (De la Rua et al., 2009; Parejo et al., 2016) and risk of foreign pests and pathogens introduction (Owen, 2017). As a result, imports are advised against by beekeeping associations (e.g. in Switzerland, press releases of beekeeping association BienenSchweiz can be found here: https://www.bienen.ch/aktuelles/detail/gefahr‐von‐bienenimporten‐575.html and here https://www.bienen.ch/aktuelles/detail/erschreckend‐hohe‐zahl‐an‐bienenimporten‐658.html; consulted on 20.12.2022), which could reduce the attractiveness and acceptance of this approach.

### 2.2 Was survival to *V. destructor* infestation of naturally selected local honey bee stocks achieved?

The survival of local *V. destructor*‐infested populations under natural selection‐based programmes has been attributed to the occurrence and selection of genetic resistance traits (Locke, 2016; Mondet, Beaurepaire, et al., 2020; Moro, Blacquière, Panziera, et al., 2021; Panziera et al., 2017). A genetic basis for the survival of two such populations is indeed likely because despite translocation to new environments, colonies still showed lower infestation levels than the local control (Schnell, 2007). In most cases, the role of resistance genes is, however, unclear and favourable environmental factors after the interruption of varroacidal treatments can explain survival, at least in part (e.g., lack of beekeeping pressure, amount and diversity of food resources [Le Conte et al., 2007]). Such factors are likely involved in the survival of untreated French populations of Avignon and Sarthe because the colonies died after translocation to a new environment (Vaublanc et al., 2003).

In a programme in which surviving populations are generated by splitting the fittest colonies (Blacquière, Boot, Calis, Moro, et al., 2019), it is possible that splitting decreased infestation levels (Haber et al., 2019) and contributed to reported survival (Moro, Blacquière, Panziera, et al., 2021; Panziera et al., 2017). Thus, this survival would be because of natural selection, beekeeping management or more likely, a combination of both. In addition, failure using this approach has been reported (Blacquière et al., 2020), indicating that the expected goal may not be achieved in every environment or with every honey bee population.

Thus, exposing local stock to the selective pressure of *V. destructor* can lead to survival because of the presence of resistance or tolerance genes, to favourable environmental conditions, to beekeeping management or to their combination. To date, there has been no recipe for how to achieve the desired goal or for the conditions that facilitate survival in every situation.

### 2.3 Are lineages from naturally selected programmes widely available?

Surviving stock obtained from natural selection programmes is rarely available on the market (European Commission, 2022; Le Conte et al., 2020; Le Conte & Mondet, 2017; Locke, 2016). In addition, the survivability of many populations that have been described as surviving infestations by *V. destructor* (e.g., Mondet, Beaurepaire, et al., 2020) has not been empirically ascertained. Beyond the persisting need for some degree of mite control for some of these stocks, a reason for their restricted availability could lie in a lack of acceptance resulting from their low or unknown conformity to behavioural or productivity expectations of beekeepers (e.g. Locke, 2016). This constraint does, however, not apply to local populations under natural selection programmes for rewilding or for conservation in a noncommercial setting. Overall, better knowledge of the context in which and processes by which truly surviving populations emerged could allow for replicating their successes in other regions towards beekeeping, conservation or rewilding goals.

## 3 ADVANTAGES AND DRAWBACKS OF LETTING NATURAL SELECTION SOLVE THE ‘VARROA PROBLEM’

### 3.1 Advantages

Harnessing natural selection to obtain populations surviving *V. destructor* infestations does not require the challenging task of identifying the locally adapted or resistance/tolerance traits underlying survival (Figure 1) and the laborious measure of their expression in the stock to be selected (Guichard et al., 2020). Whatever the underlying mechanisms are, only the outcome, that is, survival, selects the colonies used to produce the following generation (Blacquière, Boot, Calis, Moro, et al., 2019). The workload required to identify and select these surviving colonies is advantageously low, though not without technical challenges (see the drawbacks section). The undirected selection process is also advantageous because it can result in the joint selection of multiple mechanisms favouring colony survival (Figure 1), including tolerance, which would be very challenging to achieve under the directed selection programmes (Guichard et al., 2020). Natural selection may also lead to populations adapted to their current environment, even if it is different from the original natural honey bee environment, namely forests (Browne et al., 2020; Crane, 1999). This is particularly interesting for honey bee rewilding purposes because the primary forests honey bees have evolved in have almost disappeared, and their remnants are small and fragmented (Sabatini et al., 2018). In addition, re‐establishing large swaths of secondary natural, microhabitat‐rich forests in which rewilded honey bee populations could nest and thrive, as did their wild ancestors, would take over a century, given the slow regeneration process of forests (Braunisch et al., 2019; Paillet et al., 2015, 2017).

### 3.2 Drawbacks

#### 3.2.1 Implementation problems

Natural selection programmes will likely be limited by a lack of isolation from populations under a different selection regime because of the long mating range of honey bee queens and drones and the resulting long‐distance gene flow (Jensen et al., 2005; Neumann et al., 1999; Peer & Farrar, 1956). If sexuals from managed colonies are within the flight range of the population under natural selection, introgression will occur, as has been shown by attempts to conserve the genetic homogeneity of several subspecies (Pinto et al., 2014). Such long‐range gene flow will undoubtedly scramble the genotypes or gene networks required for survival (e.g. Dietemann & Locke, 2019) and, at best, delay the establishment of an equilibrium with the parasite, if not prevent it altogether. In most countries, regions in which a sufficient degree of isolation is possible are likely few.

Isolation could also limit the spread of pathogens from colonies under natural selection to neighbouring apiaries. The collapse of heavily infested susceptible colonies, which is expected in a ‘live and let die’ approach, provides opportunities for workers from healthier colonies to rob poorly defended food reserves, become infested by mites or other pathogens and vector these back to their own colonies (DeGrandi‐Hoffman et al., 2016; Frey et al., 2011; Goodwin et al., 2006; Greatti et al., 1992; Nolan & Delaplane, 2017; Sakofski, 1989; Seeley & Smith, 2015). Robbing can occur within and between apiaries over distances greater than a kilometre (Peck & Seeley, 2019; Seeley & Smith, 2015). This spread can occur even within the population under selection and undermine the process by leading to excessive infestation rates, even for resistant colonies (Dietemann & Locke, 2019; Seeley, 2017). Thus, the colonies under natural selection programmes should not be simply left to die. Natural selection programmes require a minimum time investment with frequent controls and the preventive killing or treatment of collapsing colonies (Seeley, 2017).

These interventions could, however, be counterproductive if they occur too early for those colonies that might have survived, despite a temporary drop in vitality. Determining when to intervene and interrupt a potential adaptation process to protect neighbouring colonies or apiaries is a compromise with no known optimal solution at this time. In rewilding programmes, the use of natural nests, which cannot be easily opened (e.g. hive boxes in trees; Blacquière, Boot, & Calis, 2019; trunk hives, Zeidler), precludes such controls and the danger of collapsing colonies spreading disease should be considered for the success of the project itself, as well as for the managed apiaries within flight range, if any.

After treatment interruption, colonies may survive because of favourable environmental factors, not because of heritable resistance or tolerance traits (see Guichard et al., 2020). Therefore, colonies originating from such programmes may experience elevated mortality in the case of translocation or environmental modifications (e.g. climate change or high between‐year variation of local environment) (Figure 1). Because, to date, there is no knowledge of which environmental parameters are essential for survival, it is not possible to predict in which regions environment‐based surviving populations could be translocated successfully. Such translocations may not be relevant for conservation or rewilding purposes but may be desirable for productive beekeeping operations, especially those migrating to track nectar flows or crops to be pollinated.

The loss of many susceptible colonies following the strong selection imposed by *V. destructor* infestation is also economically costly and may not be viable for all beekeeping business models (Figure 1). It should also be noted that the deliberate induction of a honey bee colony death is considered unethical and against the standards of animal welfare (World Organisation for Animal Health, 2018), with some countries considering it illegal (e.g. Germany, Bundesministerium der Justiz und für Verbraucherschutz Germany & Bundesamt für Justiz Germany, 2014; Switzerland, Conseil fédéral suisse Switzerland, 1995). Hence, such programmes could suffer from poor acceptance by beekeepers, conservationists and authorities.

#### 3.2.2 Outcome desirability

Apart from the direct cost of losing colonies, the traits expressed by surviving colonies do not necessarily match the expectations of stakeholders (Figure 1). Desirable traits may not be selectively advantageous when nature is steering the process (e.g. low colony size, Fries et al., 2006; low productivity, Le Conte et al., 2007; Locke, 2016; and high aggressiveness, Locke, 2016). This issue is not restricted to natural selection programmes, and it also limits the success of selection for *V. destructor* resistance traits (Guichard et al., 2020). However, in a context of general high attention to honey bee health, particular categories of beekeepers are inclined to sacrifice desirability, such as productivity, for the increased resilience of their stock (Guichard et al., 2019). This desirability constraint is alleviated if colonies are naturally selected for conservation or rewilding purposes that do not require particular traits beyond long‐term survival. An exception might occur regarding the aggressiveness of the population. In cases where high defensive behaviour is coselected with survivability (Locke, 2016), the spread of the population to sedentarily and densely inhabited regions may lead to conflicts (Winston, 1992).

#### 3.2.3 Genetic structure of naturally selected populations and long‐term sustainability

Because most colonies in susceptible populations succumb to *V. destructor* infestation (Rosenkranz et al., 2010), the stocks used in natural selection programmes are likely to experience drastic decreases in size within 1–3 years (Fries et al., 2006) (Figure 1). Such rapid colony losses lead to a low number of mating cycles in the population before reaching a bottleneck, which could prevent the establishment of gene associations that promote survival to infestations. After reaching this bottleneck, surviving populations could also be at risk of inbreeding, which is known to have multiple detrimental consequences for honey bee health (Brückner, 1979; Moritz, 1986; Tarpy et al., 2013; Woyke, 1976). Genetic bottlenecks constitute major limitations to the long‐term maintenance of a healthy surviving population. Honey bee populations may, however, be more resilient to a loss of genetic diversity than what has been expected to date. A comparison between museum and modern honey bee samples has indicated that populations experiencing high transitory colony losses can recover in size and genetic diversity, the latter probably fuelled by the polyandrous mating of queens (Mikheyev et al., 2015) or by the high recombination rate in the honey bee (Beye et al., 2006). The severity of bottlenecks affecting honey bee populations without threatening their sustainability remains to be determined. A deeper understanding of genetic diversity recovery in honey bees is also required to assess the minimum viable population sizes and risks and benefits of natural selection programmes.

Small populations remaining after a bottleneck are also highly susceptible to genetic drift, which may have stronger effects on genotypes than selection (Page & Laidlaw, 1982), possibly eliminating favourable *V. destructor* resistance or tolerance alleles and, hence, limiting the acquisition or fixation of host traits that allow for survival. Genetic drift being a major concern in small populations, the application of natural selection programmes for the conservation of endangered populations, such as the native subspecies of *A. mellifera* in their original range (De la Rua et al., 2009; Meixner et al., 2010; Parejo et al., 2016; Requier et al., 2019) may be seriously limited and require the utmost caution.

Bottlenecks and genetic drift can also cause the loss of favourable alleles not linked to resistance or tolerance to the parasite but that can be potentially beneficial to population resilience against future challenges such as environmental changes (Figure 1) or new or current but evolving parasites and pathogens (Hoban et al., 2021 and the next section).

The probability of achieving long‐term sustainability of surviving populations directly depends on the size and genetic diversity of the populations remaining after the expected bottleneck. This size is likely positively correlated with the initial size of the populations entered in the selection programme. Previous programmes used several dozens to hundreds of colonies—150 for Gotland (Fries et al., 2006), 70 in a the so‐called ‘black box’ experiment in the Netherlands (see below and Panziera et al., 2017) and 268 in a French programme (Kefuss et al., 2016)—but in the absence of long‐term monitoring of these populations' health, there is little certainty that this size was sufficient. The Gotland case indicates that the initial 150 colonies left untreated may have been too few. Down to a size of eight colonies at its lowest (Fries et al., 2006), this so‐called ‘Bond’ population experienced high genetic drift (Lattorff et al., 2015). This drift may have resulted in an inability of the population to cope with the increasing density of honey bee colonies on the island and potentially associated increase in drifting mites (Dietemann & Locke, 2019). The excessive *V. destructor* infestation rates measured in the Bond colonies two decades after the bottleneck prompted their treatment with an acaricide to ensure the survival of this scientifically important population (Dietemann & Locke, 2019).

#### 3.2.4 Coevolution with mites and viruses

In the case that a natural selection programme was successful and led to a host–parasite equilibrium, the dynamic process of coevolution should be considered regarding the perspectives for long‐term maintenance of the equilibrium. The adaptation of *V. destructor* in response to honey bee traits has not been considered sufficiently in directed selection programmes (Eliash & Mikheyev, 2020) and is also relevant for natural selection‐based programmes. The adaptation of *V. destructor* in response to honey bee traits may negate the benefits acquired by host adaptation to the parasite's previous traits. For example, the increase of genetic diversity of mites at the end of the beekeeping season when infestation rates increase creates variability on which natural selection can act, for instance, to provide mite resistance to acaricides (Beaurepaire, Krieger, & Moritz, 2017). New advantageous chemosensing capacities or modified mite reproduction cycles could be naturally selected in a similar manner because of their ability to cope with the complex chemical cues emitted by honey bees or their resistance behaviour (Nazzi & Le Conte, 2016).

Another drawback limiting the long‐term sustainability of populations selected via a live and let die selection is that it can favour increased *V. destructor* virulence: highly virulent mites leading to colony collapse can be transported by robbing honey bees to other hosts within their flight range, conferring the mites a selective advantage, at least in the short term (DeGrandi Hoffman et al., 2017; Dynes, Berry, Delaplane, de Roode, & Brosi, 2019; Seeley, 2017). Changes in *V. destructor* traits may indeed occur rapidly enough to affect the dynamics of the host–parasite relationship within a few years (Beaurepaire, Krieger, & Moritz, 2017; Moro, Blacquière, Dahle, et al., 2021). Such mite adaptation could result in increased colony mortality if the host cannot keep pace with the appropriate counter‐adaptations. Knowledge on the range of possible adaptations and counter‐adaptations in mites and honey bees should be acquired (Eliash & Mikheyev, 2020) to improve our ability to predict and possibly secure future gains in natural (as well as human‐directed) selection towards host–parasite equilibria.

An increased virulence of the parasite may also be driven by mutations, recombination or hybridization (Greenspan et al., 2018; King et al., 2015). Hybridization was detected between *Varroa jacobsoni* and *V. destructor* (Dietemann et al., 2019) and could threaten the original host: *A. cerana* (Lin et al., 2021). Recombination between *V. destructor* genomes can occur following mating between the offspring of different foundresses sharing a common brood cell. The frequency of multiple infested cells increases with the infestation rates of colonies (Beaurepaire, Ellis, et al., 2017). Given the high likelihood of elevated infestation rates in colonies subjected to the ‘live and let die’ approach, recombination may frequently occur.

If an equilibrium between the honey bee and current invasive *V. destructor* Korea 1–1 variant (Navajas et al., 2010) can be reached by means of natural selection, the underlying mechanisms may not allow for adaptation to a new invasive variant, haplotype or species. The effectiveness of resistance traits may vary according to the genome of the parasite (Garrido et al., 2003; Koskella, 2018). New haplotypes of not only *V. destructor*, but also *V. jacobsoni*, have already spilled over to *A. mellifera* and may further spread (Beaurepaire et al., 2015; Roberts et al., 2015); in addition, more such events could occur in the future. Similarly, the survival traits selected may not remain efficient at protecting colonies if the viruses associated with the mite became more harmful, as, for example, occurs when strains adapt to a new intermediate host (Gisder et al., 2018). A higher virulence would reduce the infestation threshold, leading to colony collapse (Le Conte et al., 2010; McMahon et al., 2016). A breakdown of the equilibrium could also occur if new strains of a virus with different characteristics were to spread in the population (Dietemann & Locke, 2019). Although highly virulent virus strains, for instance, those causing the death of the honey bee pupae or limiting the fitness of the mite (Giuffre et al., 2019) should be counter‐selected, they may cause considerable damage before their extinction or before a putative adaptation by a reduction of their virulence (Eliash & Mikheyev, 2020). This process may lead to partial or total population collapses before a new equilibrium has been reached. Such fluctuations do not represent a fundamental problem for conservation or rewilding as long as they are natural mechanisms (i.e. not triggered by humans), but would generate unpredictable harvests, endangering the sustainability of the beekeeping industry.

#### 3.2.5 Letting natural selection act in a nonnatural environment

Outside of primary forests, populations naturally selected with productivity, conservation and rewilding goals are potentially negatively affected by the highly disturbed environment (Durant & Otto, 2019; Otto et al., 2016), which does not correspond to the ecological conditions under which honey bees have evolved. In addition, the keeping of colonies in spatially restricted apiaries at a higher than natural density combined with the current colony management in productive and sometimes conservation beekeeping is unfavourable to honey bee health because it favours disease spread and increased pathogen virulence (Brosi et al., 2017). These generally more stringent conditions, especially when compared with the original natural context, might require new adaptations to arise in natural selection‐based programmes selected and untreated populations. The ability of free‐living *A. mellifera* populations, which can nest and survive at least temporarily in man‐made structures and urban environments (Browne et al., 2020; Dubaić et al., 2021), suggests that such adaptations are possible or that the honey bee is plastic enough to live in a disturbed environment. However, verifying the self‐sustainability of the currently described free‐living populations is required to ascertain this ability.

#### 3.2.6 Prospects of solving the ‘varroa problem’ by harnessing natural selection

A random population used for starting a natural selection programme may not possess the genotype necessary to ensure survival when infested by *V. destructor* or may not be placed in an environment that would enable survival. A positive outcome is not guaranteed for each and every programme, and population collapse is a risk. The few examples of population collapse published (Berg et al., 2001; Blacquière et al., 2020; Dettli, 2009) may reflect reporting bias, which prevents an accurate evaluation of the prospects for solving the varroa problem using natural selection‐based programmes. However, the many theoretical and practical challenges suggest low prospects for success. This is particularly problematic given the apparent high importance of environmental factors in the establishment of an equilibrium between the hosts and parasites. The correspondingly low genetic basis for survivability likely precludes the retention of this trait in exported stock from successful programmes. Evolutionary processes regulating host–pathogen interactions can also affect the outcome of natural selection‐based programmes. However, some of these processes can be steered in the desired direction by beekeeping management. Below, we propose strategies to overcome some of these challenges and to increase the probability of successfully and locally harnessing natural selection.

## 4 PERSPECTIVES ON HARNESSING NATURAL SELECTION TO SOLVE THE *V. DESTRUCTOR* PROBLEM

Selection strategies minimizing the emergence of virulent pathogenic strains, the number of colonies lost and the associated costs are desirable. As a perspective towards these goals, we propose strategies to improve the probability for a successful outcome in natural selection‐based programmes, also taking into account their distinct possible objectives. We have shown above that a nonnegligible degree of human intervention is required to assist the process and ensure the long‐term occurrence and effectiveness of selected traits under field‐realistic conditions at low costs and with minimal collateral damages. Thus, the proposed process involves a combination of natural and human‐directed selection processes.

### 4.1 Decreasing costs

The economic cost of losing colonies lacking the genotypes required to survive *V. destructor* infestations can be mitigated through the use of several approaches. In the ‘soft Bond’ (Cakmak & Fuchs, 2013; Kefuss et al., 2009) and ‘black box’ (Blacquière, Boot, Calis, Moro, et al., 2019) approaches, highly infested, collapsing colonies or colonies with low probability of survival are removed from the selection programme and treated, or their queens are replaced by better adapted ones and maintained in the programme. This not only allows for saving colonies as production units, but it is also ethically more acceptable because, in the worst case, only individuals (the poor‐performing queens)—not entire colonies—are killed. Depending on the type of hives used and if they allow for access to the queen, for acaricide treatments and for translocations, this strategy is also applicable for conservation or rewilding programmes.

Another alternative to letting susceptible colonies die is to identify the fittest colonies in advance. Following this ‘black box’ approach, two populations of untreated colonies were selected in the Netherlands (Blacquière, Boot, Calis, Moro, et al., 2019). Selection was based not only on high winter survival, but also on traits such as good spring development and high production of drone brood. Such early phenotyping seems possible because, in unselected colonies left without treatment, a trade‐off is generally observed between the number of drones produced and colony survival (Kraus et al., 2007). However, the appropriate or best colony trait being an early predictor of survival may not be known in advance, so it might take a few years to identify it in the population of which one would like to promote survival (see Section 4.5). The selection of such traits could follow typical human‐directed trait selection concepts based on estimated breeding values, which would allow for the optimization of genetic progress, but depend on the availability of pedigrees from herd‐book or genotype data. Here, too, hive types may limit the access to the colony required to measure such traits.

### 4.2 Avoiding collateral damage

Ethical considerations aside, having an isolated location and long distances between colonies would alleviate the need for killing or removing collapsing colonies to protect neighbouring ones (Büchler & Hoffmann, 1991), thus fully exploiting the process by giving all chances for the traits underlying survival to be selected. In addition, isolation will promote the mating of queens with drones of the test population and increase the frequency of alleles allowing selection progress and adaptation to local conditions (Szabo & Lefkovitch, 1987). Establishing mating stations on sufficiently isolated islands or mountain valleys, as well as performing artificial insemination (Page & Laidlaw, 1985), are suitable solutions.

If such a degree of isolation is not possible, removing or treating highly infested colonies before their collapse may improve the survival of neighbouring colonies by decreasing mite exchanges. To determine when the right time is to kill or remove collapsing colonies, we recommend comparing the development of individual colonies to the typical development pattern of other colonies (included treated ones) in the same environment. The criteria and their respective threshold values past which the colonies should be killed or removed have not been clearly determined to date and should be determined based on frequent observations and local beekeeping experience.

### 4.3 Optimizing genetic diversity

The starting population needs to be of a sufficient size and diversity before treatments are stopped or reduced to increase the probability of capturing genotypes favouring survival and prevent genetic drift after reaching a population bottleneck. Initial genetic diversity could be confirmed with genetic markers (Bourgeois & Beaman, 2017), but the lack of knowledge on the molecular basis of survivability (Mondet, Beaurepaire, et al., 2020) prevents measuring functional diversity. Because the level of diversity required to ensure the sustainability of a population over time is not known and depends on the extent of future environmental changes it will be subjected to, a prudent strategy would be to conserve the highest genetic diversity possible in the selected honey bee populations. The existing examples (Blacquière et al., 2020; Fries et al., 2006; Kefuss et al., 2016) indicate the need to include more than a hundred colonies in a natural selection programme. The need to recruit such diversity should, however, be balanced with that of including only locally adapted colonies, which should be available in sufficient numbers, and of the implementation costs.

The loss of genetic diversity may be reduced if the interruption of acaricide treatment is not abrupt but progressive, reducing parasitic pressure and leaving more time for adaptations to arise. This strategy was followed in a Finnish population (see www.buckfast.fi), where the quantity of oxalic acid applied per colony was decreased from year to year. The disadvantages of a progressive increase of selection pressure would be slower reaching of the end goal and acquisition of resistance against treatments by mites if they are exposed to concentrations below that required for optimal effectiveness (Benito‐Murcia et al., 2021; Odenholt et al., 2003).

Maintaining a sufficient number of backup colonies of the initial population under normal *V. destructor* management (e.g. the mother colonies of those included in the selection programme) could contribute to restoring genetic diversity following the loss of susceptible genotypes. A few treated colonies from the backup population could be introduced to the population under selection each year to recover lost alleles. Keeping backup populations of treated colonies can also secure genetic resources that may possess traits favourable against future challenges and would be advantageous in case no genes ensuring survival to *V. destructor* infestations are present in selected stock, hence leading to its extinction. This is particularly central to conservation programmes. This backup population should be distant enough from the population under natural selection to prevent constant and unwanted gene flow.

As a last resort, in case of a deleterious decrease of genetic diversity but with the risk of disrupting local adaptation (e.g. for *V. destructor* resistance traits, which may differ between populations), diversity could be increased by importing colonies from foreign surviving populations.

### 4.4 Influencing the evolution of mite virulence with beekeeping practices

The survival of honey bee colonies despite *V. destructor* infestation can be promoted if the parasite's virulence decreases. Such a decrease can be promoted by adapting apicultural practices to exploit principles of the evolution of virulence (Brosi et al., 2017; Cressler et al., 2016; Neumann & Blacquière, 2017; Read, 1994). For instance, adapting an apiary layout by separating colonies by several dozens of metres (Nolan & Delaplane, 2017; Seeley, 2017; Seeley & Smith, 2015) can significantly reduce drift and robbing (Jay, 1966a, 1966b, 1968) and, hence, horizontal transmission of parasites and pathogens between the colonies (Dynes, Berry, Delaplane, Brosi, & de Roode, 2019; Neumann & Blacquière, 2017; Nolan & Delaplane, 2017; Seeley & Smith, 2015). High intercolonial distance is likely to reduce the probability of highly virulent mites killing their colony early to reach a new host when compared with a crowded apiary (Peck & Seeley, 2019). In apiaries with more spaced colonies, mites would tend to have a lower selective advantage in killing their host colony.

Reducing beekeeping management‐induced stress by adapting beekeeping practices to ecological and evolutionary conditions favourable to the honey bee could promote the expression of host defence mechanisms and favour colony survival (Neumann & Blacquière, 2017; Seeley, 2017). However, to date, it is unclear which conditions are essential to reduce this stress (e.g. decrease management intensity, decrease apiary density and use hive types closer to natural nesting sites) in an economically sustainable way for the beekeeping industry. In contrast, such stress would be greatly alleviated in rewilding or conservation programmes if the latter were not implemented within a commercial setting.

### 4.5 Implementation: proposed selection programmes

**FIGURE 2:**
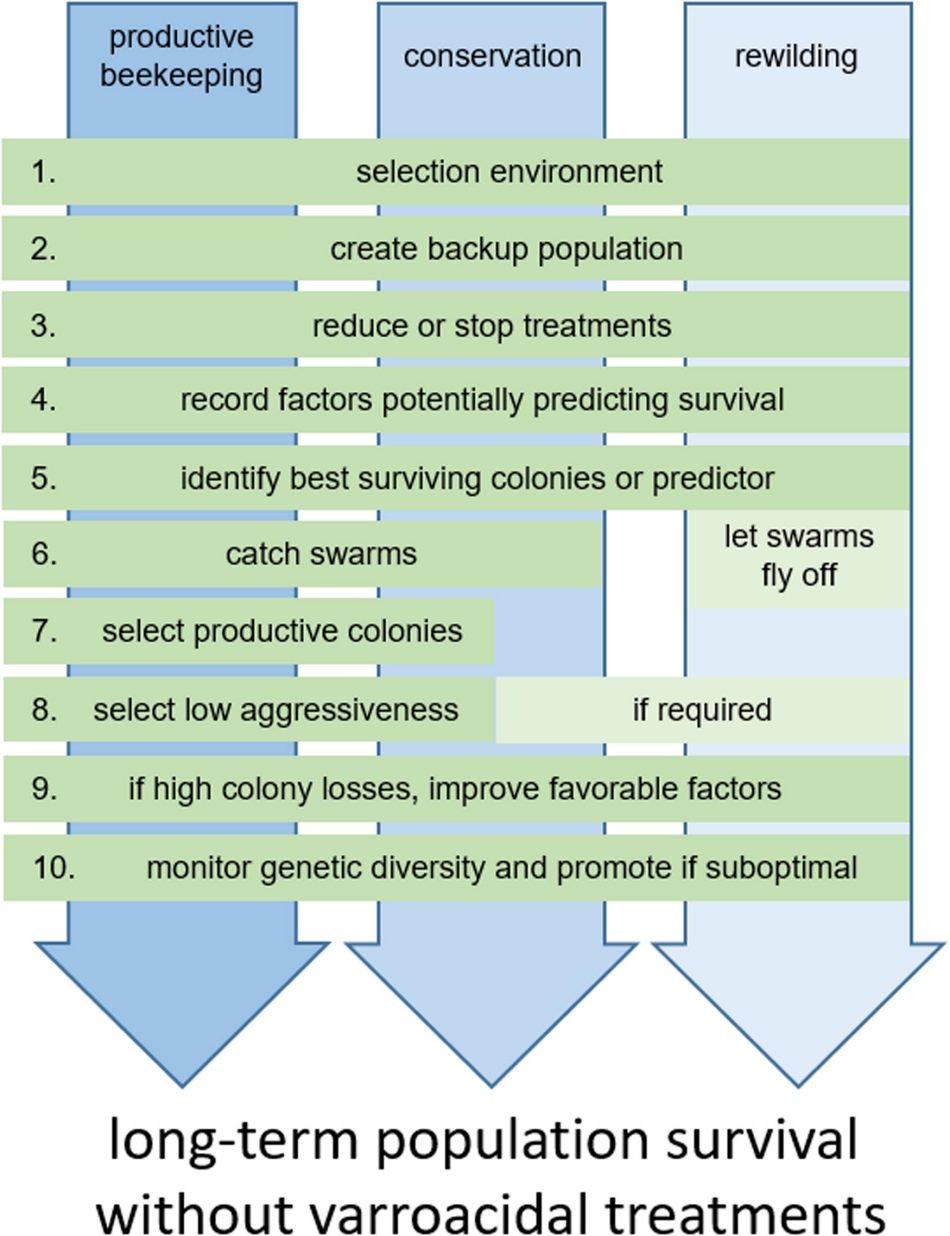
Proposed strategies for the selection of honey bee populations surviving infestations with *Varroa destructor* in absence of varroacidal treatments. The process varies according to the objective of the natural selection programme: productive beekeeping, conservation or rewilding (see the Glossary).

Harnessing natural selection to favour the survival of honey bee colonies infested by *V. destructor* could, in theory, be achieved in the framework of productive beekeeping, conservation or rewilding projects. The strategies proposed to generate increased survival to *V. destructor* in the absence of varroacides applications are presented in Figure 2. In the absence of field validation to date, they should not be interpreted as ready‐to‐implement recipes to be recommended generally but as guidelines to overcome challenges and limitations of the natural selection approach for scientists or beekeepers interested in developing a treatment‐free solution to the varroa problem. However, these strategies are not applicable in countries where foregoing *V. destructor* treatments is illegal. A change in legislation or exceptions in the frame of research projects should be sought with the relevant authorities before initiating such programmes.

Identifying an environment favourable for colony development and survival (diverse source of nutrients, suitable climate, etc.) (Asensio et al., 2016; Brodschneider & Crailsheim, 2010; Donkersley et al., 2020) and in which the population can be established is beneficial to increase chances of success (Figure 2(1)). The creation of a backup population helps limit genetic losses in cases of population collapse after treatments are stopped (2). Varroacidal treatments are then stopped or reduced on a population as large as possible (3) to apply selective force on the target colonies. During the first year, we suggest recording as many parameters as possible, which can correlate with colony survival and help predict survival probability in the next years (4). These parameters are not necessarily linked to putative *V. destructor* resistance traits (e.g. infestation rates) and could be easier to measure, such as number of drones produced, colony size at particular times, winter food consumption or occurrence of adult workers with deformed wings. The early recognition of susceptible colonies and their treatment can reduce the risk of losing them, thus reducing the collateral damage while shortening the generation time and promoting genetic progress. After the critical period for colony survival at the chosen location (e.g. winter in the temperate regions), the best surviving colonies or those with a high probability of survival can be selected for preferential breeding (5).

Differences among the goals of the programmes, which are either restricted to survival or include economic traits, determine whether swarms will be controlled and whether feeding will be provided or if desirable traits (such as productivity or reduced defensive behaviour) will be selected for (6–8). Colony reproduction by swarming should be followed through swarm capture and relocation in beekeeping or conservation programmes but left to proceed naturally without recapture in rewilding initiatives. The intensity of colony management also depends on the types of hives used, which can limit interventions for swarm control, feeding or evaluation of desirable traits in the case of natural nesting sites in rewilding programmes, for example. Feeding to compensate for a resource‐deficient environment could be envisaged for productive and conservation purposes but would not be desired for rewilding purposes. Conditional to the availability of colony pedigrees, steps 5 to 8 can benefit from the use of conventional breeding tools, such as the estimation of breeding values and heritability to promote genetic progress.

In the case of high colony losses, environmental conditions could be improved to favour colony survival, such as providing more natural nesting conditions, decreasing colony density and varying hive orientation or increasing interhive distance (9) (Dynes, Berry, Delaplane, Brosi, & de Roode, 2019; Jay, 1966b; Nolan & Delaplane, 2017; Seeley & Smith, 2015) (Figure 1). This could positively affect the outcome of the programme while minimizing the loss of genetic diversity. This loss can be prevented by a surveillance programme and, if required, counteracted by compensation measures (10), such as the addition of foreign‐resistant colonies to the selected population.

Significant differences in the proposed approach exist compared with already proposed concepts. Compared with the black box selection principle (Blacquière, Boot, Calis, Moro, et al., 2019), dissimilarities are (a) the absence of systematic splitting, which may affect the dynamics of mite infestation and swarming, events able to influence colony destiny (Fries et al., 2003; Loftus et al., 2016; Neumann & Blacquière, 2017); (b) early markers of survival are not predefined but result from observation of the population under selection; and (c) a distinction here is made for the three possible objectives of production, conservation and rewilding. It has elsewhere been suggested to first select for resistance traits to reach sufficient colony survival before letting natural selection act (van Alphen & Fernhout, 2020): in this case, trait selection—not natural selection—would lead to improved survival, while the efficacy of the approach would directly depend on the a priori choice of the traits. In our approach, we suggest letting natural selection determine which traits would be worth selecting in local populations.

## 5 CONCLUSION

If the harnessing of natural processes to select surviving colonies can alleviate the workload because of the lack of need to identify the traits allowing for survival and associated phenotype measurement, the approach is not exempt from all work and bound to many implementation challenges that are underreported or unforeseen by the literature. Some of the warnings we issue are theoretical and may not occur, but their consideration is crucial when planning a natural selection‐based programme. Given the gaps in the current understanding of adaptations within the *A. mellifera*–*V. destructor*–viruses system, natural selection programmes may or may not favour colony survival after an undefined number of years. This uncertainty and the timescale involved should be considered when starting a selection programme. The outcome of a natural selection‐based programme depends on local environmental conditions, on the proportion of resistant colonies already present in the population or on the success of reciprocal adaptation during the selection process. As a result, the probability of obtaining stock surviving *V. destructor* infestation in the absence of acaricidal treatments cannot be predicted. Once achieved, the long‐term maintenance of the survival of the selected population is uncertain. This maintenance can be subjected to coevolutionary changes, such as those resulting from counter‐adaptation in the pathogen (Burdon et al., 2014), from the spread of new pathogen species, haplotypes or variants (Grindrod et al., 2021; Kevill et al., 2021; Natsopoulou et al., 2017) or, more generally, from environmental changes (Le Conte & Navajas, 2008). This is especially significant if the population goes through a severe bottleneck during the selection process. In addition to the unpredictability of its outcome, natural selection may lead to unwanted outcomes, limiting its potential to solve the *V. destructor* problem. Hence, human intervention to decrease collateral damage during the selection process, optimize genetic diversity and ensure the desirability of the surviving population for beekeepers of human neighbours is required in most cases. These precautions will increase the probability of success and acceptance of natural selection‐based programmes. In addition to the possibility of taking advantage of conventional breeding tools to promote the survivability and desirability of the selected stock, these programmes would also benefit from an increased understanding of the factors contributing to success or failure. A lack of reports of natural selection‐based programmes in the literature prevents a full drawing on past experience, be it positive or negative. Negative results are notoriously neither readily nor easily published in the literature. Furthermore, such programmes may be driven by beekeepers without scientific knowledge or supervision to ensure the recording and publishing of their outcomes in a way that could benefit the community. As for resistance trait selection programmes (Guichard et al., 2020), collaborations between researchers and beekeepers are a likely avenue for optimal progress when attempting to harness natural selection to increase honey bee survival to *V. destructor* infestations.

## GLOSSARY

**Productive beekeeping**: beekeeping type, for which the commercialisation of hive products (honey, pollen, royal jelly, etc.) or pollination services is a main goal.

**Conservation programmes** aim at safeguarding local native subspecies or ecotypes. Although such programmes could be implemented on nonmanaged populations, they are usually led with populations managed by beekeepers and exploited for hive products production or pollination (De la Rua et al., 2009; Parejo et al., 2016).

**Rewilding** programmes aim at reintroducing honey bee colonies in nature to recover nonmanaged free‐living populations. This reflects the situation predating the invasion of *Varroa destructor*, which, together with the changes in land use that led to the lack of natural nesting sites and natural areas, led to the disappearance of wild populations in most regions of the Northern hemisphere where *Apis mellifera* was native. These can be implemented using standard hives as used commercially or hives designed to replicate natural nesting sites (e.g., trunk hives, Zeidler hives).

In practice, these categories are not mutually exclusive, and several may be followed simultaneously (e.g., productive beekeeping operations using local honeybees towards their conservation), possibly leading to compromises in the desired traits for the honeybee population used.

**Trait selection** aims at increasing the frequency of a trait or traits of interest (e.g., productivity, low swarming tendency, pathogen resistance) in the population through a human‐directed selective breeding of colonies showing the desired properties.

**Natural selection‐based programmes** are also driven by beekeepers or breeders but aim at increasing survival in a population exposed to the selection pressure of interest (here *V. destructor*) without favouring particular traits apart from survivorship. The current approach does not rely on the usual breeding tools (e.g., calculation of breeding values, heritability estimation) and represents a paradigm change between humans determining the traits to be selected to leaving this to natural selection.

## CONFLICT OF INTEREST STATEMENT

The authors state that they have no conflicting interests.

## Data Availability

Our perspective is based on a review work, so we do not have associated data to archive.
